# Adaptive replanning intensity-modulated radiotherapy for choroidal metastasis of breast cancer using optical coherence tomography

**DOI:** 10.1093/jrr/rru023

**Published:** 2014-04-04

**Authors:** Toshihiko INOUE, Norihisa MASAI, Ryoong-Jin OH, Hiroya SHIOMI, Noriyasu HASHIDA

**Affiliations:** 1 Miyakojima IGRT Clinic, 1-16-22 Miyakojima-ku, Osaka, 534-0021, Japan; 2 Department of Ophthalmology, Osaka University Graduate School of Medicine, 2-2 Yamadaoka, Suita, 565-0871, Japan

**Keywords:** adaptive radiotherapy (ART), intensity-modulated radiotherapy (IMRT), optical coherence tomography (OCT), choroidal metastasis

## Abstract

Swept source optical coherence tomography (SS-OCT) is a convenient method for precise, early-stage detection of choroidal metastatic lesions, involving assessment of tumor response, and for regular follow-up studies. Using information obtained with SS-OCT, we performed intensity-modulated radiotherapy (IMRT) for a patient with choroidal metastasis from breast cancer with more accuracy than had been previously possible. We made replanning adaptive radiotherapy (ART) three times based on the rapid tumor shrinkage detected by weekly assessments with SS-OCT. Accordingly, the planning target volume (PTV) decreased from 1.6 cm^3^ to 0.61 cm^3^ (38%), with 0.95 cm^3^ (59%) and 0.75 cm^3^ (46%) as intermediate values during the treatment course. The D_0.1 cm3_ of the right optic nerve was also reduced from 1.70 Gy/fraction to 0.69 Gy/faction, with 1.41 Gy/fraction and 1.29 Gy/fraction as intermediate values. Adaptive replanning IMRT made it possible to perform locally curative treatment of the metastatic choroidal lesion with a higher dose for the PTV, and a lower dose for organs at risk (OARs).

## INTRODUCTION

Definitive radiotherapy generally has only a limited role in the treatment of primary and metastatic tumors in the orbital region. There are very few diseases of the orbital region for which curative radiotherapy is indicated, these being: (i) choroidal melanoma, treated with proton beam therapy or brachytherapy using ^60^Co disc and ^125^I plaques, (ii) retinoblastoma, treated with electron or photon beam therapy, and also brachytherapy, and (iii) pediatric rhabdomyosarcoma, treated with external radiotherapy [1–5]. Sophisticated immobilization procedures are essential with these treatments in order to achieve good treatment results in terms of visual outcome.

On the other hand, recent advances in technology have enabled the treatment of detailed features of tumors near highly radiosensitive or risky structures. Image-guided radiotherapy (IGRT) is a technology offering hopes for the future. When a precision image for localization of the corresponding tumor itself is obtained, adaptive radiotherapy (ART) can be introduced. In the field of ophthalmological oncology, it has been found possible to use swept source optical coherence tomography (SS-OCT) for precise examination of the choroid [6]. It has been found possible to perform locally curative radiotherapy for metastatic choroidal diseases using advanced OCT. This is the first time that metastatic breast cancer in the choroid has been treated with adaptive replanning intensity-modulated radiotherapy (IMRT) using precision OCT imaging.

The main purpose of this paper is to demonstrate clearly the usefulness of SS-OCT for performing locally curative ART for a metastatic choroidal lesion, which is not possible to be evaluated by CT and MRI.

## MATERIALS AND METHODS

### Case presentation

The patient was a 51-year-old female with advanced right breast cancer. Seven years previously, she had developed a large mass in the right axillary fossa. A physician then diagnosed advanced breast cancer, but the patient refused the standard treatment of chemotherapy followed by radical mastectomy, and chose immunotherapy. Two and a half years later, she developed multiple bone metastases in the spine and pelvis, which were treated successfully with goserelin acetate (Zoladex^®^), zoledronic acid (Zometa^®^) and anastrozole (Arimidex^®^). Four years after the initial diagnosis, she underwent transcatheter arterial chemoembolization with epirubicin-loaded superabsorbent polymer microspheres for massive lesions of the chest wall, and axillary and mediastinal lymph nodes [7]. Five years and 10 months after the initial diagnosis, she developed migraines and complained of double vision with decreased right visual acuity; she was referred to our clinic, and was found to have paresis of right cranial nerves II to IX. She then underwent IMRT for metastatic lesions of the right cavernous sinus, sphenoid bone and cerebellopontine angle (detected with magnetic resonance imaging (MRI)). The treatment was carried out with the prescribed dose of 20 Gy in two fractions followed by 30 Gy in 10 fractions over a total of 20 days. Recovery from the right cranial nerve paresis was shown 2 months later, and bone involvement had completely disappeared after 11 months.

**Fig. 1.**
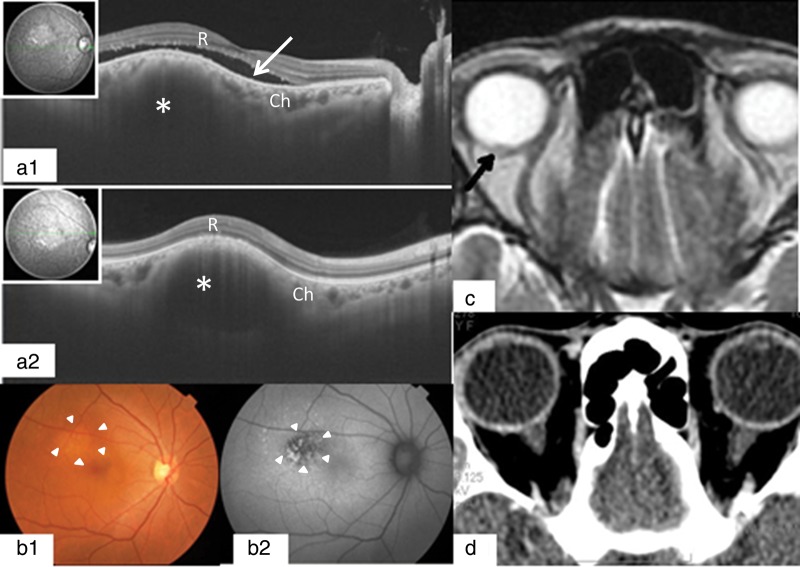
OCT images at the first visit showed a metastatic lesion of the choroid (Ch) (*), dome-shaped elevation of the neurosensory retina (R) and retinal pigment epithelium, with adjacent subretinal fluid (white arrow) (**a**). Fundus photographs and autofluorescence images of the right eye showed subretinal masses and retinal pigment epithelial damages (arrowheads) due to choroidal infiltration of the tumor (**b**). MRI showed a small change (black arrow) in the right retina–choroidal area near the right optic nerve (**c**). However, CT did not give abnormal findings in the same part (**d**).

During July 2012, the patient again showed decreased right visual acuity. In October 2012, an ophthalmological oncologist, using SS-OCT, at the Department of Ophthalmology in the Osaka University Hospital, detected a very small metastatic lesion in the right choroid, associated with serous retinal detachment. The SS-OCT findings were that there was a light-colored tumor in the subretinal region of the superior-temporal portion of the right macula. The size of the tumor was two disc diameters. Subretinal fluid was present around the tumor, and some irregularities of the retinal pigment epithelium (RPE) were found. It was possible, retrospectively, to detect a very small metastatic choroidal lesion with MRI, but this was not detected with CT at the time (Fig. 1). Ophthalmologic examination showed decreased right visual acuity (RV = 0.8; LV = 1.5) without change in the visual field.

### Equipment

The equipment used was as follows: (i) treatment device: 6-MV X-ray Novalis unit^TM^ (BrainLAB AG, Germany); (ii) treatment planning system: BrainSCAN^TM^ and iPLAN RT Dose ver.4.1.2^TM^ (BrainLAB AG, Germany); (iii) CT-simulator: BrightSpeed^TM^ (GE, USA); (iv) MRI-simulator: SIGNA HDx^TM^ (GE, USA); (v) immobilization device: Vac-Lok cushions^TM^ and Thermoplastics (CIVCO, USA); and (vi) image-guided radiotherapy system: ExacTrac^TM^ X-ray positioning system and 6-axis robotic couch (BrainLAB AG, Germany). Movement of the patient's eyeballs was evaluated using the cine-mode MRI obtained at the first treatment planning.

## RESULTS

### Parameters of adaptive replanning IMRT

**Fig. 2.**
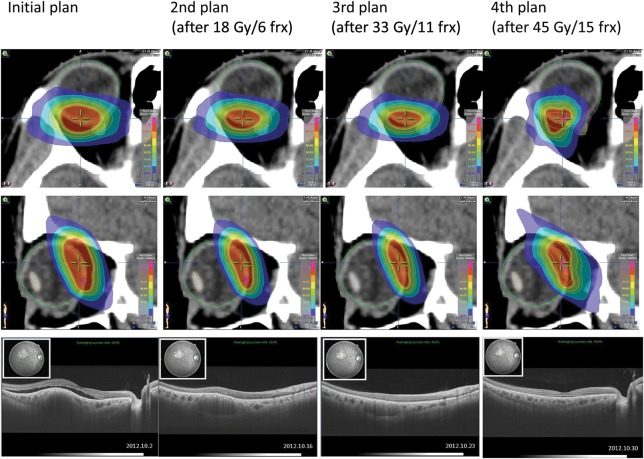
Adaptive replanning IMRT for right choroidal metastasis, based on CT images in axial view (top line) and sagittal view (middle line), in which GTV was defined using information from the SS-OCT image (bottom line). By the fourth plan, the monitor unit was changed to avoid overdoses to the right optic nerve.

**Fig. 3.**
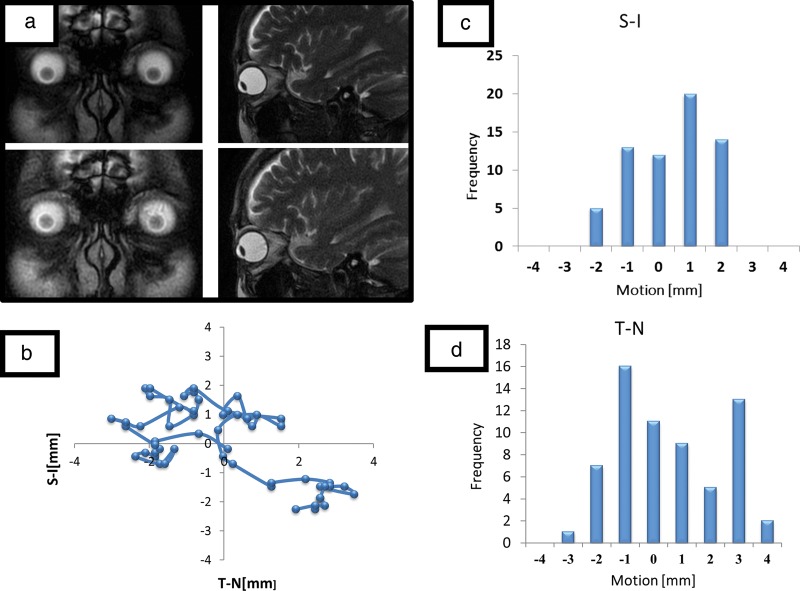
FIESTA 2D cine-mode MRI examination of the eyeballs was carried out, without a shell and with closed eyes, to assess the size of the internal margin (IM) (**a**). A track of the lens motion was prepared with 30-s serial images at 0.5 s/frame (**b**), and the positional error of the lens was 0 ± 1.3 mm in the vertical direction (S–I), and 0 ± 1.9 mm in the horizontal direction (T–N) (**c**, **d**).

The gross tumor volume (GTV) was determined by combining SS-OCT data and CT-MRI fusion images. Treatment of the patient's choroidal metastasis was initiated with non-coplanar 7-beam IMRT using 6-MV X-ray Novalis^®^ (Fig. 2). The initial planning target volume (PTV) was 1.6 cm^3^, which was decided upon on the basis of a 3-mm margin added to the internal target volume (ITV). We prescribed a dose of 60 Gy in 20 fractions based on the previous IMRT for the metastatic lesion of the right sphenoid bone. We generally adopt the standard BED10 (biologically effective dose of α/β = 10) of 80 Gy for definitive radiotherapy at our clinic. Dose–volume histogram (DVH) parameters of PTV were chosen as D_95%_ > 95%, V_95%_ > 95%, and V_107%_ < 3%. Dose constraints of OARs (organs at risk) were as follows: for the right eyeball, D_0.1 cm3_ < 107%, D_0.5 cm3_ < 105%, D_1 cm3_ < 100% and D_5 cm3_ < 30%; for the right optic nerve, D_0.1 cm3_ < 70%, and D_0.5 cm3_ < 10%; and for the right lens, D_max_ < 5%. The patient underwent FIESTA 2D cine-mode MRI examination of the eyeballs, with no shell and with closed eyes, to determine the magnitude of the internal margin (IM). A track of lens motion was prepared with 30-s serial images at 0.5 s per frame. The positional error of the lens was 0 ± 1.3 mm in the vertical direction (S–I) and 0 ± 1.9 mm in the horizontal direction (T–N) (Fig. 3). On this basis, during treatment (with a shell and with closed eyes), the internal margin (IM) was estimated to be 3 mm. The mean treatment time was 13 min per session.

The clinical target volume (CTV), which was equal to the GTV, was estimated as detailed above. During the 23-d treatment course, the PTV was reduced three times because of volume decrease, based on the SS-OCT data. We could estimate the localization of the small metastatic lesion in the right choroid with correlation to the macula, e.g., direction and distance from the center of the macula. However, we could not make an on-line fusion image with CT and SS-OCT because of the lack of a coordinated system between both images. For instance, the thickness of the macula is 250 μm (0.25 mm); on the other hand, the slice thickness of our planning CT is 1.25 mm. Such a discrepancy in the scale makes it difficult to perform precision radiotherapy for orbital lesions at the present time. Accordingly, our strategy of replanning ART was performed by means of an offline method. The PTV was reduced to 0.95 cm^3^ (59%) at the first reduction (after six fractions), then at the second reduction (after 11 fractions) to 0.75 cm^3^ (46%), and at the third reduction (after 15 fractions) to 0.61 cm^3^ (38%). We describe the reduction in the size of the PTV and the ITV (according to the GTV data obtained from SS-OCT during the treatment course) in more detail as below. At the second step of ART (after six fractions), we observed a GTV reduction of 0.007 30 cm^3^ (66%). However, we postponed the ITV reduction, based on our clinical experience of some unexpected marginal recurrence. Accordingly, we reduced the PTV margin from 3 mm to 2 mm at the time of second planning, based on the assurance of the set-up margin over the previous six treatment sessions. So the PTV was reduced 0.95 cm^3^ (59%). At the third step (after 11 fractions), the ITV was reduced to 0.10 cm^3^ (53%), and the PTV was reduced to 0.75 cm^3^ (46%), based on the GTV reduction to 0.00310 cm^3^ (28%). At the fourth step (after 15 fractions), we observed a rapid decrease in the GTV of 0.00016 cm^3^ (1.5%). We decided on the dose constraint by the right optic nerve to be a D_max_ of 1.65 Gy/fraction (54%), with a partial volume decrease in the PTV adjacent to the optic nerve. The size of the PTV decreased to 0.61 cm^3^ (38%). At the last reduction, the monitor unit of each beam was changed in order to avoid late injury to the optic nerve [8, 9]. Although the initially planned prescribed dose was 60 Gy in 20 fractions over 4 weeks, treatment was completed after 17 fractions because of the rapid tumor disappearance observed with SS-OCT. Therefore, D_0.1 cm3_ (one of the DVH parameters) of the right optic nerve decreased from 1.70 Gy/fraction to 0.69 Gy/fraction, with 1.41 Gy/fraction and 1.29 Gy/fraction as intermediate values. By means of IMRT, however, we could precisely control the dose to the right optic nerve, which was located in close proximity to the GTV. Using 3D-conformal radiotherapy (CRT), we could not have obtained such a complicated dose constraint to the OARs in the vicinity of the GTV (Table 1). However, repetitive ART did not reduce the dose as estimated at the center of the macula because the lesion was located too close to the macula in this case. Fortunately, we could reduce the actual dose to the macula from the planning dose of 60 Gy to the given dose of 51 Gy, based on the significant evidence of tumor clearance with SS-OCT.

**Table1.** Change of DVH parameters by repetitive ART

	Initial plan	2nd plan	3rd plan	4th plan	Cumulative Dose(Gy)	
					Initial plan	ART
Prescribed Dose (Gy/Frx)	18/6	15/5	12/4	6/2	60/20	51/17
PTV (cm^3^)	1.62	0.95	0.75	0.61		
ITV (cm^3^)	0.19	0.19	0.10	0.10		
GTV (cm^3^)^a^	0.011	0.0073	0.0031	0.00016		
Rt. Optic nerve (Vol. 0.69 cm^3^)						
D_max_ (Gy/Frx)	3.03	2.88	2.79	1.65	60.6	47.0
D_mean_ (Gy/Frx)	0.72	0.62	0.59	0.39	14.4	10.6
D_0.1 cm3_ (Gy/Frx)	1.70	1.41	1.29	0.69	34.0	23.8
D_0.5 cm3_ (Gy/Frx)	0.10	0.06	0.06	0.07	2.0	1.3
Rt. Eyeball (Vol. 8.19 cm^3^)						
D_max_ (Gy/Frx)	3.24	3.24	3.21	3.30	64.8	55.1
D_mean_ (Gy/Frx)	1.02	0.84	0.75	0.88	20.4	15.1
D_0.5 cm3_ (Gy/Frx)	3.09	2.98	2.89	2.83	61.8	50.7
D_1.0 cm3_ (Gy/Frx)	2.84	2.49	2.27	2.23	56.8	43.0
Rt. Lens (Vol. 0.12 cm^3^)						
D_max_ (Gy/Frx)	0.06	0.06	0.03	0.09	1.2	1.0
D_mean_ (Gy/Frx)	0.04	0.03	0.03	0.07	0.8	0.7
D_0.1 cm3_ (Gy/Frx)	0.01	0.01	0.01	0.03	0.2	0.2
Rt. Choroid (Vol. 3.35 cm^3^)						
D_max_ (Gy/Frx)	3.24	3.24	3.21	3.27	64.8	55.0
D_0.1 cm3_ (Gy/Frx)	3.16	3.17	3.14	3.12	63.2	53.6
D_0.5 cm3_ (Gy/Frx)	2.86	2.62	2.48	2.21	57.2	44.6
D_1.0 cm3_ (Gy/Frx)	1.59	1.15	1.01	1.07	31.8	21.5
Rt. Macula						
Point dose at center (Gy/Frx)	3.15	3.18	3.15	3.09	63.0	53.6

GTV (cm^3^)^a^ estimated with SS-OCT. D_max_ = maximum dose, D_mean_ = mean dose, D_0.1 cm3_ = minimum dose delivered to 0.1 cm^3^, D_0.5 cm3_ = minimum dose delivered to 0.5 cm^3^. D_1.0 cm3_ = minimum dose delivered to 1.0 cm^3^, Frx = fraction.

**Fig. 4.**
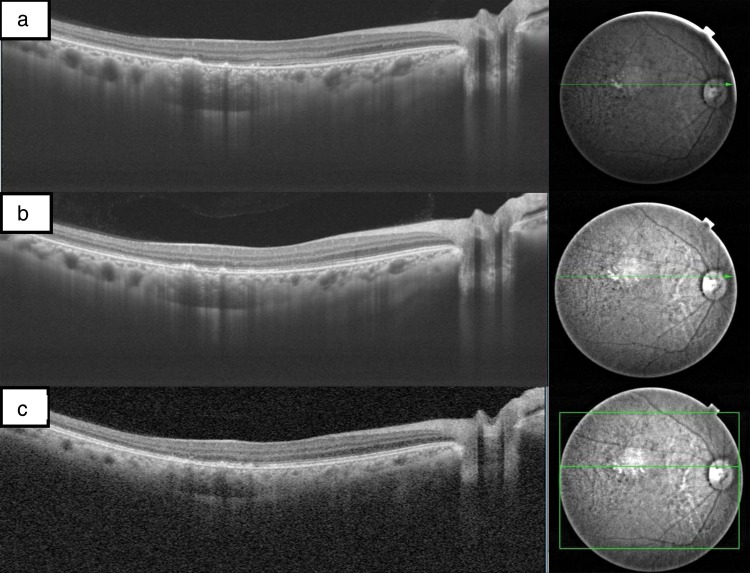
A follow-up study with SS-OCT showed complete disappearance of the choroidal lesion by 6 days after ART (**a**), and a complete response 1.5 and 9 months later (**b**, **c**).

On the sixth day after completion of adaptive replanning IMRT, SS-OCT revealed no visible tumors in the choroid. By December 2012, the choroidal tumor had completely disappeared, and the patient had recovered visual acuity (RV = 1.5; LV = 1.5). OCT then revealed normal vascular structures in the choroid and some residual irregularities in the RPE, without subretinal fluid. As of August 2013, the patient had maintained the improved visual acuity (RV = 1.5; LV = 1.5), with the same OCT findings as previously (Fig. 4).

## DISCUSSION

Half a century ago, replanning was carried out in clinical practice for lung treatment involving bronchial atelectasis, and esophageal treatment involving prestenotic dilatation. In those cases, the tumors were found to shift markedly from the original locations as a result of the effects of treatment during the early courses of radiotherapy. For prompt detection of these tumor shifts, we identified indices such as minor changes in the clinical symptoms, including sputum discharge with necrotizing masses or little alleviation of dysphagia. We also performed weekly checks with X-ray imaging for the replanning (as a type of ART in a broad sense).

As a result of recent advances in technology, treatment plans can now be changed readily during the routine practice of sophisticated precision radiotherapy. ART is a process for adjusting treatment plans in response to changes during the radiotherapy treatment course. In the case of radiosensitive tumors, deformations of targets and normal structures (as well as of the patient anatomy) are frequently found, and the treatment plan must be adjusted for each deformation. ART includes different levels for different clinical situations. According to the National Radiotherapy Implementation Group Report, ART is included in IGRT complexity and is classified at four levels (from 4a, which is a strategy of replanning with offline imaging, to 4d, which is real-time (4D) ART) [10]. State-of-the-art 4D-ART with autocontouring and autoplanning could be an appropriate method for adjusting the set-up error in each treatment session. One of the essential aims of this technique is to increase the tumor dose by reducing the PTV, and thus to reduce the dose reaching surrounding OARs. Therefore, the most important tool for ART is imaging technology, such as CT, MRI and PET-CT. Until 10 years ago, in the field of ophthalmologic oncology, the precise location of the GTV was not determined with the above-mentioned imaging or fundoscope, despite OCT having become commercially available for clinical use 16 years ago. However, the most recent technical innovations enable determination of the precise anatomic structures of the retina and choroid, and minute lesions in the choroid can now be measured using SS-OCT [6].

## CONCLUSION

In conclusion, it was found to be possible to perform weekly checks of metastatic choroidal lesions using SS-OCT. As a result, we succeeded in replanning three times during the 20-fraction course of IMRT in the original treatment planning. The repetitive ART did not reduce the dose estimated at the center of the macula, because the lesion was located too close to the macula. However, the decision was made to discontinue the treatment after 17 fractions of IMRT, and it was possible to spare the dose of 9 Gy in three fractions. As a result, the D_0.1 cm3_ of the right optic nerve decreased from 34.0 Gy to 23.8 Gy in total. It was found possible to carry out adaptive replanning IMRT for metastatic choroidal lesions using SS-OCT. At the present time, it is not possible to make a definitive report about multiple replanning ART [10], but changes in treatment parameters were made in every session during the time-series presented here, and the technical improvements in SS-OCT as described should contribute to locally curative precision radiotherapy for orbital lesions in the future.
